# Urinary retention on an acute geriatric hospitalisation unit: prevalence, risk factors and the role of screening, an observational cohort study

**DOI:** 10.1007/s41999-021-00495-3

**Published:** 2021-04-18

**Authors:** Katleen Fagard, Kasper Hermans, Mieke Deschodt, Sofie Van de Wouwer, Frank Vander Aa, Johan Flamaing

**Affiliations:** 1 Department of Geriatric Medicine, University Hospitals Leuven, Herestraat 49, Box 7003 35, 3000 Leuven, Belgium; 2 Division of Gerontology and Geriatrics, Department of Public Health and Primary Care, KU Leuven, Leuven, Belgium; 3 Division of Rheumatology, Department of Internal Medicine, Maastricht University Medical Center, Maastricht, The Netherlands; 4 Care and Public Health Research Institute (CAPHRI), Maastricht University, Maastricht, The Netherlands; 5 Healthcare and Ethics, Faculty of Medicine and Life Sciences, UHasselt, Hasselt, Belgium; 6 Department of Endocrinology, GZA Hospital, Campus Gasthuiszusters, Antwerp, Belgium; 7 Department of Urology, University Hospitals Leuven, Leuven, Belgium

**Keywords:** Urinary retention, Post-void residual volume, Bladder scan, Prevalence, Risk factors, Elderly

## Abstract

**Purpose:**

Urinary retention (UR) is common in older patients. The aim of this observational cohort study was to measure the prevalence of UR in patients aged ≥ 75 years on admission to an acute geriatric hospitalisation unit and to determine which at risk group would benefit from screening.

**Methods:**

Post-void residual volumes (PVR) were measured within 3 days of admission with an ultrasound bladder scan. Uni- and multivariable analysis were used to determine risk factors associated with PVR ≥ 150 and ≥ 300 millilitres.

**Results:**

Ninety-four patients, mean age 84.6 years, were included. The male/female ratio was 0.7. Patients with PVR ≥ 150 (29.8%) had more urological comorbidities, symptoms of overflow incontinence, voiding difficulties, subtotal voiding, faecal impaction, urinary tract infection (UTI) and were more frequently referred because of urinary symptoms. Patients with PVR ≥ 300 lived less at home, had more urological comorbidities, dysuria, voiding difficulties, subtotal voiding, constipation, faecal impaction, UTI, detrusor relaxants, and were more frequently referred because of urinary symptoms. Voiding difficulties and referral because of urinary symptoms were independently associated with PVR ≥ 150. Not living at home, reporting subtotal voiding, constipation, and referral because of urinary symptoms were independently associated with PVR ≥ 300.

**Conclusion:**

Screening for UR on admission to an acute geriatric hospitalisation unit is most indicated in patients with urinary and defaecation problems. However, because the prevalence was high, because UR was also observed in patients without these problems, and history taking may be difficult, the threshold for PVR measurement in acutely ill geriatric patients should be low.

**Trial registration:**

Clinicaltrials.gov NTC04715971, January 19, 2021 (retrospectively registered).

**Supplementary Information:**

The online version contains supplementary material available at 10.1007/s41999-021-00495-3.

## Key Summary points

**Aim:**

To determine the prevalence of urinary retention and the role of screening for urinary retention on admission to an acute geriatric hospitalisation unit.

**Findings:**

A post-void residual volume (PVR) ≥150 ml was present in 29.8% of patients and was independently associated with reporting voiding difficulties or referral to the hospital because of urinary symptoms. A PVR ≥300 was present in 16.0% of patients and was independently associated with not living at home, reporting subtotal voiding, having constipation, and referral to the hospital because of urinary symptoms.

**Message:**

Screening for urinary retention is most indicated in patients with urinary and defaecation problems, but a low threshold for screening is recommended in all acutely ill geriatric patients

**Supplementary Information:**

The online version contains supplementary material available at 10.1007/s41999-021-00495-3.

## Introduction

Urinary retention (UR) is the inability to empty the bladder completely and can be acute or chronic [1]. The International Continence Society defines acute UR as a painful, palpable or percussible bladder in which the patient is unable pass any urine, while chronic UR is defined as a non-painful bladder, which remains palpable or percussible after the patient has passed urine [2].

Diagnosing UR in older patients may be difficult. Symptoms and clinical signs may be less clear and older patients often present with atypical symptoms, such as immobility, falls, delirium, loss of appetite or failure to thrive [3–5]. Moreover, chronic UR may be accompanied by overflow incontinence [2]. As a consequence, UR may go unnoticed [6]. This can lead to various complications of the lower urinary tract, such as recurrent urinary tract infections (UTI), incontinence, detrusor dysfunction, haematuria, bladder stones, and of the upper urinary tract, such as hydroureteronephrosis, renal dysfunction, and urosepsis [7, 8].

Because some of the abovementioned complications may lead to increased morbidity and even mortality, UR should be diagnosed correctly and in a timely manner. As such, it is important to identify high-risk groups who would benefit from systematic screening for UR. Some of the risk factors that have been described in studies are male gender, cognitive impairment, diabetes, faecal impaction, urinary incontinence, history of previous prostate, bladder or voiding problems, history of neurological disorders, immobility and the use of anticholinergic medications [9, 10]. Limited research has been conducted regarding indications for portable ultrasound bladder screening. Moreover, standardized cut-offs for clinically significant post-void residual volumes (PVR) are lacking [9–11].

After diagnosing UR, it is important to address the underlying causes that are often multifactorial. Causes can be divided into the following categories: outflow obstruction, infection or inflammation, drug-induced, detrusor underactivity, neurological and other causes (including postoperative and psychogenic UR) [12–14]. Often there is a combination of underlying causes or ‘predisposing factors’ and factors that trigger UR or ‘precipitating factors’, such as UTI, faecal impaction, excessive fluid intake or diuretics, alcohol, trauma, anaesthesia, pain, immobilisation, and certain medications [14–19]. The more ‘predisposing factors’, the less ‘precipitating factors’ are required to develop UR, and vice versa. This explains why older patients with multimorbidity need less noxious insults to develop UR.

Few studies examined the prevalence of UR in older patients on admission. Most studies were conducted in internal medicine units [11, 20] or rehabilitation centres [9, 10, 21]. Grosshans et al. (1993) were the only ones including older patients on admission to an acute geriatric hospitalisation unit [22]. They reported a PVR ≥ 150 millilitres (ml) in 26%, ≥ 300 ml in 12%, and ≥ 500 ml in 6% of patients. To our knowledge, no studies have studied whom best to include in systematic bladder scan screening for UR on admission to an acute geriatric hospitalisation unit.

The aim of this study was to assess the prevalence of UR in patients aged 75 and over admitted to an acute geriatric hospitalisation unit and to determine which patients would benefit most from systematic portable ultrasound bladder screening on admission.

## Methods

### Sample and setting

An observational cross-sectional cohort study was conducted on the acute geriatric hospitalisation units of the University Hospitals Leuven in Belgium which have a collective capacity of 80 beds. Admission criteria for these wards are defined in the Belgian care programme for geriatric patients: the average age of patients should be ≥ 75 years and they should be in need of a geriatric multidisciplinary approach for one or more of the following reasons: a frailty profile; active polypathology; limited homeostasis; atypical clinical disease presentation; disturbed pharmacokinetics; risk of functional decline; risk of malnutrition; tendency to be inactive or bedridden, with an increased risk for institutionalisation and for dependency in activities of daily living; psychosocial problems [23].

All patients aged 75 years or older admitted with or without an indwelling urinary catheter between October 26 and December 18, 2015 were consecutively screened for inclusion within 72 h of admission. Patients with urinary catheters for any other reason than UR, patients with ascites, dialysis, uro- or nephrostomy, terminally ill patients, and postoperative patients were not eligible for inclusion. Patients who refused to participate, who were unable to understand and carry out the instructions for the PVR measurement, with a urinary catheter for chronic UR, who were discharged or died during the inclusion process were excluded. The study was approved by the Ethics Committee Research of the University Hospitals Leuven (mp08305). The study nurse (SV) obtained a written informed consent from all patients who agreed to participate in the study, or from a proxy when the patient was incapable to provide written informed consent.

### Procedures

Prior to the start of the study, information sessions were organised for the nurses from the acute geriatric hospitalisation units, in which the study nurse (SV) explained the aim of the study and the data collection procedure. In addition, information was given on the use of the portable ultrasound bladder scan and the execution of a bladder scan.

The nurses were asked to perform 3 consecutive post-void bladder scans as soon as possible after admission and at the latest within 72 h of admission as standard of care during the study period. The highest value of the 3 consecutive PVR measurements was recorded. The study nurse (SV) was present on Monday, Wednesday and Friday morning, performed informed consents and performed bladder scanning in patients where this had not yet been done. Before performing the bladder scan, the patients were asked to urinate. Whenever possible, patients had to walk to the toilet in the bathroom, with or without assistance, meanwhile their mobility was assessed.

Consecutively, the study nurse performed a bedside assessment. A mini-cog examination was performed and urinary symptoms, defaecation habits, and fall history were assessed by patient or caregiver interview using a structured questionnaire (SV). The demographic and remaining clinical data were collected from the patient’s electronic medical records (SV, KH, KF).

### Variables

#### Outcome variables

The PVR was measured using a portable ultrasound bladder scan (Verathon BladdersScan BVI 3000). Jalbani et al. found a high correlation (*r*^2^ = 0.97) between urinary catheterisation (gold standard) and bladder scanning and concluded that the use of the Verathon BladderScan BVI 3000 is as accurate as urinary catheterisation for measuring the PVR [24]. As there are no official definitions or guidelines on what is considered a relevant PVR for UR, in this study we defined UR as a PVR of ≥ 150 ml. In addition, we also included a PVR ≥ 300 ml as secondary outcome. The cut-offs were chosen for the following reasons: the first bladder-filling sensation is felt at a volume of 150 ml. At a volume of 300 ml, the pressure receptors in the bladder wall will be activated, which creates a sense of fullness of the bladder and a need to urinate [25]. Moreover, in clinical practice a PVR ≥ 300 ml is often considered as the threshold for insertion of a urinary catheter.

#### Demographics

The following patient characteristics were recorded: age, gender and living situation. Patients living alone at home and together at home were considered as living at home and patients living in an assisted living facility or nursing home were considered as not living at home. Patients living in a convent were considered as not living at home when they were functionally or cognitively impaired.

#### Comorbidities

Various comorbidities were retrospectively retrieved from the patient’s electronic medical records: neurological, gynaecological and urological history, a history of diabetes type 1 or 2, recurrent falls (more than 1 fall episode in the last 6 months), heart failure and chronic kidney disease (CKD). CKD was defined as a glomerular filtration rate (eGFR, calculated using the CKD-EPI formula) of < 60 ml/min/1.73 m2 [26]. An age-adjusted definition (< 45 ml/min/1.73 m^2^) was also considered [27].

#### Medication

For each patient, the anticholinergic burden (ACB) score was calculated taking into account medication use in the last 24 h before the PVR measurement [28, 29]. Antidepressants with ACB score ≥ 2, as well as antipsychotics, opioids, and detrusor relaxants were examined separately for their associations with UR. Antiepileptics, histamine H1 receptor antagonists, anti-diarrhoea medications, decongestive drugs, spasmolytics, muscle relaxants, and antiparkinsonian agents were not considered individually. Low prevalence of these drugs precluded reliable statistical analysis.

#### Clinical evaluation

Urinary incontinence was assessed by means of 3 questions: Do you have/does the patient have (1) an urge to urinate and involuntary loss of urine before reaching the toilet? (2) involuntary loss of urine when sneezing, coughing, lifting or moving something? (3) loss of small amounts or drops of urine without urge to urinate or loss of small drops of urine when changing position? Answering positive on these questions was classified as urge, stress or overflow incontinence, respectively. Dysuria was questioned as having a burning sensation or pain when urinating. Urinary frequency was questioned as urinating more often than usual. Voiding difficulty was questioned as straining to void. Subtotal voiding was questioned as the feeling of incomplete bladder emptying. Hypogastric pain was questioned as pain in the lower abdomen.

Constipation was defined as the absence of stools for 3 or more days before the PVR measurement, or if patients reported having had less than 3 stools in the last week with a need to strain and mostly hard or lumpy stools, or if the patient had faecal impaction [30]. Faecal impaction was defined as a large mass of compacted faeces in the rectum or colon on clinical or radiographic examination, that could not be spontaneously evacuated [31].

The cognitive status of the patients was evaluated using the Mini-Cog [32]. The Mini-Cog is a brief cognitive test that involves an assessment of an older person's ability to memorise three words, to draw a clock, and to recall the three words. Repeating only 1 or 2 of the 3 words and being unable to draw a clock correctly or not being able to repeat any of the words was considered as cognitive impairment [32].

Mobility was assessed by walking to the bathroom and going to the toilet. All patients were asked to urinate on the toilet prior to the PVR measurements. Patients were considered mobile if they could walk back and forth to the toilet and if they were able to stand up from the toilet independently. The use of mechanical aids was permitted. Needing assistance from another person or not being able to walk was defined as impaired mobility.

#### Laboratory evaluation and urinalysis

The patient's renal function was evaluated by serum creatinine level and eGFR (calculated using the CKD-EPI formula).

A urinary dipstick test was performed in all patients. In patients with positive leucocyte esterase or positive nitrites on the dipstick test, further urinalysis was performed. An automated leucocyte count of ≥ 10 leukocytes/mm^3^ and significant microscopic bacteriuria of > 100.000 CFU/ml for which antibiotics were started by the treating physician was considered as UTI in this study [33]. If the patient was taking antibiotics at the time of the urine sample and cultures remained negative, the presence of UTI was determined based on the leucocyte count.

#### Reason for referral to the hospital

The reasons for referral to the hospital were divided into the following categories: confusion, falls or immobility, urinary symptoms, cardiopulmonary symptoms, digestive symptoms, fever or non-specific symptoms with biochemical inflammation (leucocytosis and elevated C-reactive protein) on admission, other. In patients with multiple reasons for admission, each reason was scored separately.

### Statistical analysis

Descriptive and comparative statistics were used. Normally distributed continuous variables were reported as means with standard deviations (SD). Not normally distributed continuous variables were reported as medians with interquartile ranges (IQR). Categorical variables were reported as numbers and percentages. Baseline characteristics and clinical variables of patients with a PVR of < 150 ml versus ≥ 150 ml and a PVR of < 300 ml versus ≥ 300 ml were compared using unpaired t-tests for normally distributed continuous variables. Pearson Chi-squared tests or Fisher’s exact tests (if ≥ 1 cell had an expected count of less than 5) were used for dichotomous or nominal variables and Mann–Whitney *U* tests for not normally distributed continuous variables or ordinal variables. All tests were 2-tailed, assuming a 5% significance level. Multivariable logistic regression models were used to determine independent predictors for a PVR ≥ 150 ml and a PVR ≥ 300 ml. Significant variables in the univariable analysis were considered in a forward stepwise logistic regression procedure. P-values (Likelihood ratios), odds ratios (OR), and 95% confidence intervals (CI) are reported. The software package used was SPSS version 20 (SPSS Inc., Chicago, IL).

## Results

### Description of the sample

**Fig. 1:**
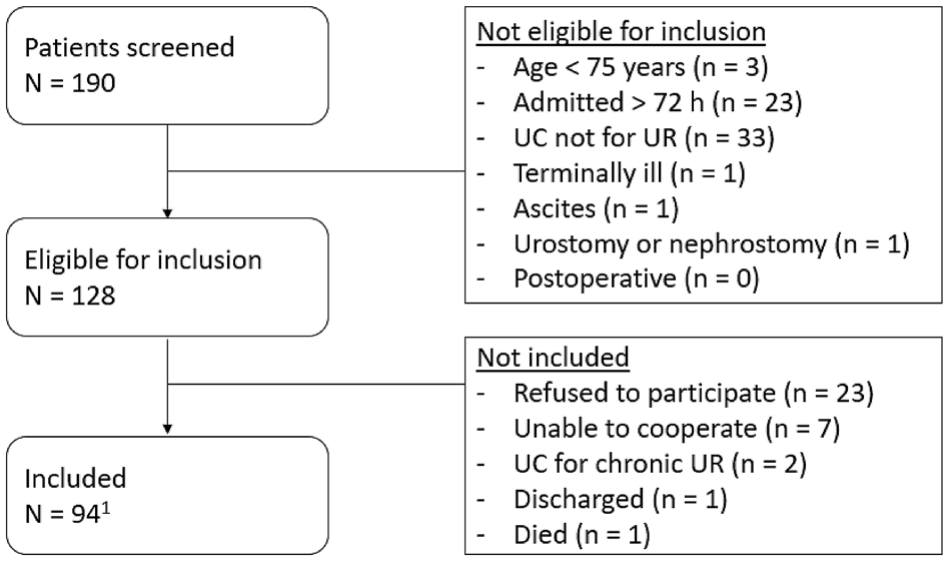
Flow-chart of in- and exclusions. *N* number; *h* hours; *UC* urinary catheter; *UR* urinary retention. ^1^patients who received a urinary catheter for UR in the emergency department were also included because their PVR was noted in their electronic medical record (*n* = 2)

Of the 190 patients admitted to acute geriatric hospitalisation units in the study period, 128 patients were eligible for inclusion (Fig. 1). The final sample included 94 patients of whom 41 were men (43.6%) and 53 women (56.4%). Of these patients, 92 were admitted directly through the emergency department. One patient was admitted directly from home, and one patient was referred and transferred from the geriatric day hospital. The mean age of the included patients was 84.6 years (range 75–100) and 76.6% of them lived at home. The median length of stay was 12 days (IQR 7–17.25).

### Prevalence of urinary retention

UR (PVR ≥ 150 ml) was diagnosed in 28 (29.8%) of the 94 patients included. Fifteen patients (16.0%) had a PVR of ≥ 300 ml and nine patients (9.6%) had a PVR between 500 and 770 ml. There were no major differences between men and women and among the different age groups (Table 1).

**Table 1:** Post-void residual volume measurements by gender and by age-group

PVR (ml)	Total, *n* (%) *N = *94	Male, *n* (%) *N = *41	Female, *n* (%) *N = *53	75–79 y, *n* (%) *N = *15	80–84 y, *n* (%) *N = *31	85–89 y, *n* (%) *N = *31	≥ 90 y, *n* (%) *N = *17
0–49	47 (50.0)	15 (36.6)	32 (60.4)	5 (33.3)	15 (48.4)	18 (58.1)	9 (52.9)
50–99	13 (13.8)	7 (17.1)	6 (11.3)	2 (13.3)	5 (16.1)	3 (9.7)	3 (17.6)
100–149	6 (6.4)	6 (14.6)	0 (0)	3 (20.0)	1 (3.2)	1 (3.2)	1 (5.9)
150–199	5 (5.3)	2 (4.9)	3 (5.7)	1 (6.7)	2 (6.4)	2 (6.4)	0 (0)
200–299	8 (8.5)	5 (12.2)	3 (5.7)	2 (13.3)	3 (9.7)	3 (9.7)	0 (0)
300–399	3 (3.2)	1 (2.4)	2 (3.8)	0 (0)	1 (3.2)	0 (0)	2 (11.8)
400–499	3 (3.2)	1 (2.4)	2 (3.8)	0 (0)	1 (3.2)	1 (3.2)	1 (5.9)
≥ 500	9 (9.6)	4 (9.8)	5 (9.4)	2 (13.3)	3 (9.7)	3 (9.7)	1 (5.9)

*N* number; *ml*: millilitres; *PVR*: post-void residual volume; *y*: years

### Risk factors associated with urinary retention

Table 2 shows potential risk factors for UR and their association with PVR ≥ 150 ml and PVR ≥ 300 ml (univariable analysis). The group of patients with a PVR ≥ 150 ml had more urological comorbidities (*P* = 0.022), more symptoms of overflow incontinence (*P* = 0.025), more voiding difficulties (*P* < 0.001), more feeling of incomplete voiding (*P* = 0.036), more faecal impaction (*P* = 0.016), more UTI’s (*P* = 0.040), and were more frequently referred to the hospital because of urinary symptoms (*P* = 0.008). The group of patients with a PVR ≥ 300 ml were more frequently not living at home (*P* = 0.040), had more urological comorbidities (*P* = 0.036), more dysuria (*P* = 0.006), more voiding difficulties (*P* = 0.006), more feeling of incomplete voiding (*P* = 0.003), more constipation (*P* = 0.009), more faecal impaction (*P* = 0.015), more UTI’s (*P* = 0.013), was more frequently taking medication for an overactive bladder (*P* = 0.050) and were more frequently referred to the hospital because of urinary symptoms (*P* = 0.002). Other causes of admission, such as confusion, falls or immobility, cardiopulmonary symptoms, digestive symptoms, or infection/inflammation were not related to UR. Neither did we find an association with the use of anticholinergic medication (other than detrusor relaxants), opioids, or loop diuretics in the last 24 h before the PVR measurement.

**Table 2:** Patient and clinical characteristics and association with post-void residual volumes (PVR ≥ 150 and ≥ 300)

	PVR ≥ 150 ml			PVR ≥ 300 ml		
	NO *N = *66	YES *N = *28	*P* value	NO *N = *79	YES *N = *15	*P* value
**Demographics**						
Age, mean (± SD)	84.8 (± 5.9)	84.2 (± 5.2)	0.638	84.5 (± 5.6)	85.1 (± 6.1)	0.713
Male gender, *n* (%)	28 (42.4)	13 (46.6)	0.720	35 (44.3)	6 (40)	0.758
Not living at home, *n* (%)	13 (19.7)	9 (32.1)	0.192	15 (19.0)	7 (46.7)	0.040*
**Comorbidities**						
Neurological, *n* (%)	27 (40.9)	14 (50)	0.416	33 (41.8)	8 (53.3)	0.408
Gynaecological, *n* (%)	12 (18.2)	4 (14.3)	0.770	15 (19.0)	1 (6.7)	0.454
Urological. *n* (%)	19 (28.8)	15 (53.6)	0.022*	25 (31.6)	9 (60.0)	0.036*
Diabetes, *n* (%)	16 (24.2)	6 (21.4)	0.768	20 (25.3)	2 (13.3)	0.508
Heart failure, *n* (%)	10 (15.2)	2 (7.1)	0.500	10 (12.7)	2 (13.3)	1.000
Recurrent falls^1^	23 (34.8)	15 (53.6)	0.091	31 (39.2)	7 (46.7)	0.591
CKD, eGFR < 60, *n* (%)	35 (53.0)	19 (67.9)	0.184	42 (53.2)	12 (80.0)	0.054
CKD, eGFR < 45, *n* (%)	23 (34.8)	11 (39.3)	0.682	27 (34.2)	7 (46.7)	0.356
**Medication**						
ACB score, media *n* (IQR)	2 (1.0–3.0)	1.5 (0–3.8)	0.693	2 (1.0–3.0)	2 (1.0–5.0)	0.428
Antipsychotics with ACB score ≥ 1, *n* (%)	7 (10.6)	5 (17.9)	0.333	8 (10.1)	4 (26.7)	0.096
Antipsychotics with ACB score ≥ 2, *n* (%)	6 (9.1)	4 (14.3)	0.478	7 (8.9)	3 (20.0)	0.196
Antidepressants with ACB score ≥ 2, *n* (%)	5 (7.6)	4 (14.3)	0.443	7 (8.9)	2 (13.3)	0.632
Opioids, *n* (%)	17 (25.8)	6 (21.4)	0.655	19 (24.1)	4 (26.7)	1.000
Loop diuretics, *n* (%)	11 (16.7)	8 (28.6)	0.189	13 (16.5)	6 (40.0)	0.072
Detrusor relaxants, *n* (%)	3 (4.5)	3 (10.7)	0.358	3 (3.8)	3 (20.0)	0.050*
**Clinical evaluation**						
Incontinence, *n* (%)^3^	36 (55.4)	20 (71.4)	0.147	46 (59.1)	10 (66.7)	0.577
Urge symptoms^4^	23 (35.4)	9 (33.3)	0.851	25 (32.1)	7 (50.0)	0.230
Stress symptoms^4^	17 (26.2)	7 (25.9)	0.982	20 (25.6)	4 (28.6)	0.754
Overflow symptoms^4^	12 (18.5)	11 (40.7)	0.025*	20 (25.6)	3 (21.4)	1.000
Dysuria, *n* (%)^4^	4 (6.2)	6 (22.2)	0.059	5 (6.4)	5 (35.7)	0.006*
Urinary frequency, *n* (%)^4^	20 (30.8)	9 (33.3)	0.810	24 (30.8)	5 (35.7)	0.759
Voiding difficulty, *n* (%)^4^	3 (4.6)	11 (40.7)	< 0.001*	8 (10.3)	6 (42.9)	0.006*
Subtotal voiding, *n* (%)^4^	8 (12.3)	9 (33.3)	0.036*	10 (12.8)	7 (50.0)	0.003*
Hypogastric pain, *n* (%)^4^	4 (6.2)	3 (11.1)	0.414	4 (5.1)	3 (21.4)	0.069
Constipation, *n* (%)^3^	13 (20.0)	10 (35.7)	0.107	15 (19.2)	8 (53.3)	0.009*
Faecal impaction, *n* (%)^3^	4 (6.2)	7 (25.0)	0.016*	6 (7.7)	5 (33.3)	0.015*
Cognitive impairment, *n* (%)	44 (66.7)	20 (71.4)	0.651	53 (67.1)	11 (73.3)	0.768
Impaired mobility, *n* (%)	39 (59.1)	22 (78.6)	0.070	48 (60.8)	13 (68.7)	0.054
**Laboratory evaluation and urinalysis**						
Creatinine, median (IQR)	1.0 (0.8–1.5)	1.2 (0.9–1.6)	0.524	1.0 (0.8–1.5)	1.3 (0.9–1.8)	0.304
eGFR, mean (± SD)	55.6 (± 22.2)	52.1 (± 24.3)	0.492	55.8 (± 21.7)	48.1 (± 27.5)	0.234
UTI, *n* (%)^5^	9 (15.0)	9 (34.6)	0.040*	11 (15.5)	7 (46.7)	0.013*
**Reason for referral to the hospital**						
Confusion, *n* (%)	14 (21.2)	7 (25.0)	0.687	16 (20.3)	5 (33.3)	0.313
Falls – immobility, *n* (%)	27 (40.9)	13 (46.4)	0.621	34 (43.0)	6 (40.0)	0.827
Urinary symptoms, *n* (%)	2 (3.0)	6 (21.4)	0.008*	3 (3.8)	5 (33.3)	0.002*
Cardiopulmonary symptoms, *n* (%)	18 (27.3)	11 (39.3)	0.249	24 (30.4)	5 (33.3)	1.000
Digestive symptoms, *n* (%)	17 (25.8)	6 (21.4)	0.655	20 (25.3)	3 (20.0)	1.000
Infection – Inflammation^2^, *n* (%)	11 (16.7)	3 (10.7)	0.543	12 (15.2)	2 (13.3)	1.000
Other, *n* (%)	18 (27.3)	3 (10.7)	0.078	21 (26.6)	0 (0.0)	0.020*

*ACB* anticholinergic burden scale; *CKD* chronic kidney disease; *ED* emergency department; *IQR* inter quartile range; *m* month; *ml* millilitres; *N* number; *PVR* post-void residual volume; *SD* standard deviation; *UTI* urinary tract infection

^1^More than 1 fall episode in the last 6 months

^2^Fever or non-specific symptoms with biochemical inflammation (elevated leucocytosis and elevated C-reactive protein) on admission

^3^Missing data in 1 patient (*N = *93)

^4^Missing data in 2 patients (*N = *92)

^5^Missing data in 8 patients (*N = *86)

*Statistical significance (defined as *P* value ≤ 0.05)

Multivariable analysis showed that reporting voiding difficulties and referral to the hospital because of urinary symptoms were significant independent predictors for PVR ≥ 150 ml (Table 3). Not living at home, reporting subtotal voiding, having constipation, and referral to the hospital because of urinary symptoms were significant independent predictors for PVR ≥ 300 ml (Table 4).

**Table 3:** Multivariable analysis (PVR ≥ 150 ml)

	Forward stepwise logistic regression	
	OR (95% CI)	*P* value
Voiding difficulty, *n* (%)	13.2 (3.2–55.3)	0.000
Referral because of urinary symptoms, *n* (%)	7.8 (1.3–47.8)	0.026

*CI* confidence interval; *eGFR* glomerular filtration rate; *ml* millilitres, *OR* odds ratio; *PVR* post-void residual volume

**Table 4:** Multivariable analysis (PVR ≥ 300 ml)

	Forward stepwise logistic regression	
	OR (95% CI)	*P* value
Not living at home, *n* (%)	20.2 (2.8–145.7)	0.003
Subtotal voiding, *n* (%)	23.3 (3.0–178.4)	0.002
Constipation, *n* (%)	16.7 (2.7–105.1)	0.003
Referral because of urinary symptoms, *n* (%)	47.0 (3.6–616.5)	0.003

*CI* confidence interval; *ml*: millilitres, *OR*: odds ratio; *PVR*: post-void residual volumes

A substantial amount of patients with UR, 8.0% for PVR ≥ 150 ml and 7.1% for PVR ≥ 300 ml, did not have urinary symptoms, UTI or defaecation problems.

## Discussion

This study examined the prevalence of UR in patients aged 75 years and over admitted to an acute geriatric hospitalisation unit and investigated factors associated with the occurrence of UR. UR defined as a PVR ≥ 150 ml was observed in 29.8% of patients. Reporting voiding difficulties and referral to the hospital because of urinary symptoms were independently associated with this outcome. UR defined as a PVR ≥ 300 ml was observed in 16.0% of patients. Not living at home, reporting subtotal voiding, having constipation, and referral to the hospital because of urinary symptoms were independently associated with a PVR ≥ 300 ml.

In comparison to other studies reporting comparable PVR cut-off values in hospitalised older patients, one in a geriatric hospitalisation unit [22] and one in an internal medicine unit [11], the prevalence or UR in our study was slightly higher: 29.8% versus 26.0% and 25.6%, respectively, for a PVR ≥ 150 ml and 16.0% versus 12.0% and 12.5%, respectively, for a PVR ≥ 300 ml. Studies in rehabilitation centres showed PVR values ≥ 150 ml in 11.4% and 18.5% of patients [9, 21] and PVR values ≥ 300 ml in 11.8% of patients [21]. The higher prevalence in our study may be due to the fact that the patients were triaged in the emergency department before admission to the acute geriatric hospitalisation unit. Triage was based on the presence of acute illness in combination with a geriatric profile, i.e. requiring a multidisciplinary approach. As such, our study population may include more vulnerable and more acutely ill patients. Another reason for higher volumes may be that we considered the highest value of 3 consecutive PVR measurements.

Factors associated with UR were mainly of urological nature: a urological history, reporting of urinary symptoms, UTI, and referral to the hospital because of urinary symptoms. Furthermore, there was an association with constipation and faecal impaction. Not living at home was associated with a PVR ≥ 300 ml. Previous studies in older patients, both performed in rehabilitation units, have also described male gender, diabetes, neurological comorbidities, cognitive impairment, immobility, and the use of anticholinergic medications as risk factors [9, 10]. These factors were studied in this study, but we did not find a significant association with UR. Although benign prostatic hyperplasia is known as the most common obstructive cause of UR, surprisingly, there were no major differences in the prevalence of UR between men and women in our study [12, 34]. UR in women is probably more frequently related to detrusor failure than to outflow obstruction [35], though we did not perform systematic urodynamic evaluations as part of this study. Differences in findings may be due to differences in definitions. For example, the study by Borrie et al. (2001) considered diabetes diagnosed for more than 15 years, while our study included all patients with diabetes, regardless of onset or organ damage [9]. Cognitive impairment and impaired mobility were assessed within 72 h of admission by a Mini-Cog test and by the ability to walk to the toilet, whereas Wu et al. (2005) performed a Mini-Mental State Examination and used the Functional Independence Measure, an 18-item tool to score mobility [10]. Delirium has been described as a risk factor for UR [4]. Our study only assessed the presence of cognitive impairment on admission and referral to the hospital because of confusion. We did not differentiate between underlying dementia and delirium. Moreover, patients who were unable to understand and carry out the instructions for the PVR measurement were excluded and therefore we may have excluded patients with severe delirium or dementia. In terms of medication, only detrusor relaxants were found to be associated with a PVR ≥ 300 ml. Higher overall scores on the anticholinergic burden scale and individual drugs with anticholinergic properties (such as antidepressants, antipsychotics and opioids), and loop diuretics were not associated with UR, which is unexpected [18, 36]. This might be attributable to multiple factors. Firstly, we did not differentiate between acute and chronic UR, as the differentiation between acute and chronic UR in older patients is not always clear and many patients present atypically. We, therefore, chose for a pragmatic approach, namely a PVR measurement in all patients regardless of symptoms. Furthermore, we did not include postoperative UR. This is important to note, as most of the current knowledge on UR and pharmaceutical risk factors stems from studies performed in patients with acute UR or in a postoperative setting. Finally, the limited sample size and relatively low prevalence of these medications in our study cohort may also have contributed to the absence of statistically significant associations.

Based on our results, screening for UR on admission to an acute geriatric hospitalisation unit would be most indicated in patients with urinary problems, patients with constipation, patients taking detrusor relaxants and patients living in a nursing home or an assisted living facility. However, many older patients have cognitive impairment (68.1% of patients in this study) which hinders the reporting of symptoms and comorbidities. The same is true for acutely ill older patients. Moreover, a substantial amount (7 to 8%) of patients with UR in this study did not have urinary symptoms, UTI or defaecation problems. Therefore, and also because of the high prevalence or UR, we think that the threshold to perform a bladder scan in acutely ill geriatric patients should be low.

There are some limitations to this study. First, it is a cross-sectional study, which only allows us to explore associations between the variables, but cannot prove causality. Second, the PVR measurements were carried out by various nurses. This may have an impact on the reliability of our measurements. On the other hand, the study was preceded by training the nurses and three repeated measurements were done. Third, the study sample is small and it is a single centre study, so subsequent multicentre studies in larger patient populations are needed to confirm the observed associations. Fourth, some variables were retrieved from the patients’ electronic medical records which can impact the quality and reliability of the data.

Further research should focus on determining clinically relevant cut-offs for PVR measurement and evidence-based guidelines for bladder management in these patients, as there is no consensus at present. It is possible that due to longstanding overstretching of the bladder, higher PVR volumes may still be considered normal in older patients and might not need immediate catheterisation. This specifically applies to asymptomatic patients, when no signs of impaired renal function, hydroureteronephrosis or other associated complications are present [6].

In conclusion, UR is a common problem among patients aged 75 and over admitted to an acute geriatric hospitalisation unit. Its presence has shown to be associated with urological and defaecation problems. However, in our opinion screening with ultrasound bladder scanning upon admission should not be limited to these patients. In view of the high prevalence, we need to be vigilant for UR at all times.

## Supplementary Information

Below is the link to the electronic supplementary material.

Supplementary file1 (DOCX 30 kb)

## Data Availability

Data are available upon contacting the corresponding author.

## Declarations

### Reporting

The manuscript reporting adheres to the STROBE guidelines for cross-sectional studies (https://www.equator-network.org/reporting-guidelines/strobe/). The STROBE checklist is included in Additional file 1.
